# Perspective: rethinking polymer crystallization pathways—two decades after Strobl’s mesomorphic-to-granular-to-lamellar model

**DOI:** 10.1140/epje/s10189-026-00629-0

**Published:** 2026-09-03

**Authors:** Christoph Schick, Wenbing Hu

**Affiliations:** 1 Institute of Physics and Competence Centre CALOR, University of Rostock, Albert-Einstein-Str. 23-25, 18051 Rostock, Germany; 2 State Key Laboratory of Coordination Chemistry, School of Chemistry, Nanjing University, Xianlin Ave. 163, Nanjing, 210023 China

## Abstract

The 2000 perspective article by Gert Strobl, “From the melt via mesomorphic and granular crystalline layers to lamellar crystallites: A major route followed in polymer crystallization?”, proposed a revolutionary multistep mechanism for polymer crystallization from the quiescent melt, fundamentally challenging the dominant single-step models of the time. Two decades later, this perspective revisits Strobl’s conceptual framework to assess its legacy, its limitations, and its continuing relevance to modern polymer science. We examine the three-stage pathway—mesomorphic layer formation, cooperative transition to a granular crystalline layer, and block merging into lamellar crystallites—through the lens of subsequent experimental, theoretical, and simulation advances. While the model’s central premise of intermediate states has stimulated extensive research, key elements remain debated. The distinction between crystal-fixed and crystal-mobile polymers has emerged as a critical factor, reinterpreting the stabilization phenomena Strobl observed and challenging the universality of his proposed pathway. Simulation studies have provided complementary insights into precursor states; yet, the direct detection of the granular crystalline layer and the mesomorphic precursor remains elusive. We identify open questions regarding the molecular origins of early stage ordering, the factors determining lamellar thickness, and the universality of granular crystalline states. Strobl’s model was important not because it provided definitive answers, but because it forced the field to confront its assumptions and inspired a generation of research that continues to advance our understanding of polymer crystallization.

**Graphical abstract:**

**Figure:**
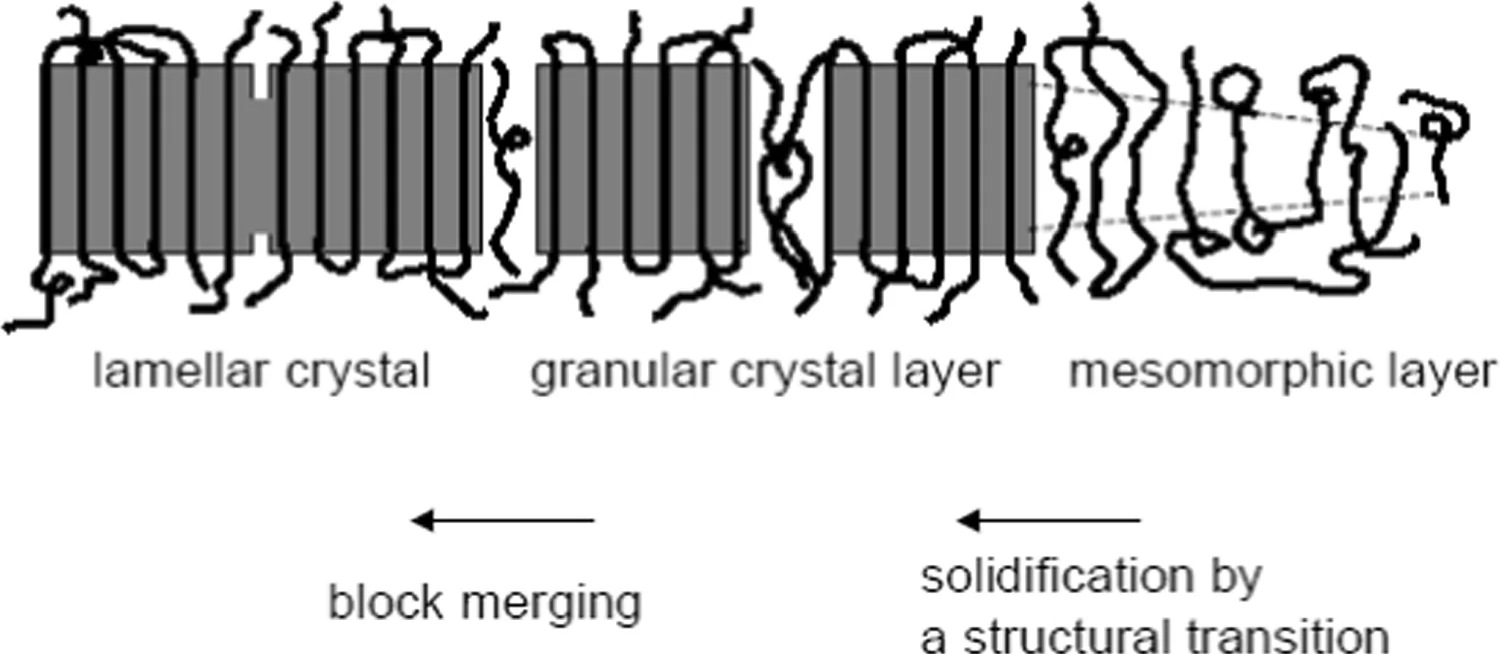

## Introduction

In the 2000 EPJE paper, ”From the melt via mesomorphic and granular crystalline layers to lamellar crystallites: A major route followed in polymer crystallization?” [1], Gert Strobl proposed a multistep mechanism for polymer crystallization from the quiescent melt, fundamentally challenging the dominant single-step models of the time. Polymer crystallization has long been one of the most fascinating and challenging problems in soft condensed matter physics [2–5]. Unlike small-molecule crystallization, which typically yields well-ordered, three-dimensional crystals with predictable thermodynamic behavior, polymers crystallize into semicrystalline structures composed of lamellae, amorphous regions, tie chains, and a complex hierarchy of defects. This structural complexity arises from the interplay between chain connectivity, entanglements, conformational freedom, and kinetic constraints. As a result, polymer crystallization has historically resisted simple theoretical descriptions, and the field has evolved through a series of conceptual frameworks, each capturing part of the phenomenon but leaving important questions unresolved. A thorough understanding of polymer crystallization is important given the immense quantity of semicrystalline polymers in modern everyday life. Currently, global polymer and plastic production amounts to approximately 435–460 million metric tons per year, with key semicrystalline polymers such as polyethylene and polypropylene accounting for over 60% of this total volume [6]. Not only is the volume of semicrystalline polymers impressive, but so are the associated challenges regarding recyclability, degradability, and processability in modern technologies such as additive manufacturing—all of which depend on crystallization processes that require careful consideration.

For much of the 20th century, the Hoffman–Lauritzen model provided the dominant conceptual foundation [7]. It offered a compelling explanation for the temperature dependence of growth rates and the characteristic lamellar thicknesses observed in semicrystalline polymers. The model assumed that lamellae grow directly from the melt via secondary nucleation on smooth growth faces, with the rate controlled by the free energy barrier required to form the first stem of a new layer at the growth front. This framework successfully rationalized many experimental observations and became deeply embedded in the interpretation of crystal growth kinetics.

However, even at the height of its influence, the Hoffman–Lauritzen model faced persistent challenges [8]. Some experiments suggested that growth faces were not atomically smooth, leading to alternative approaches such as Sadler’s entropic barrier model [9]. Nevertheless, these approaches retained the same foundational assumption: Crystallization was a one-step process in which lamellae formed directly from the entangled melt. These anomalies accumulated over decades, hinting that the growth of lamellar crystals might be more complex than a single nucleation-and-growth step.

**Fig. 1:**
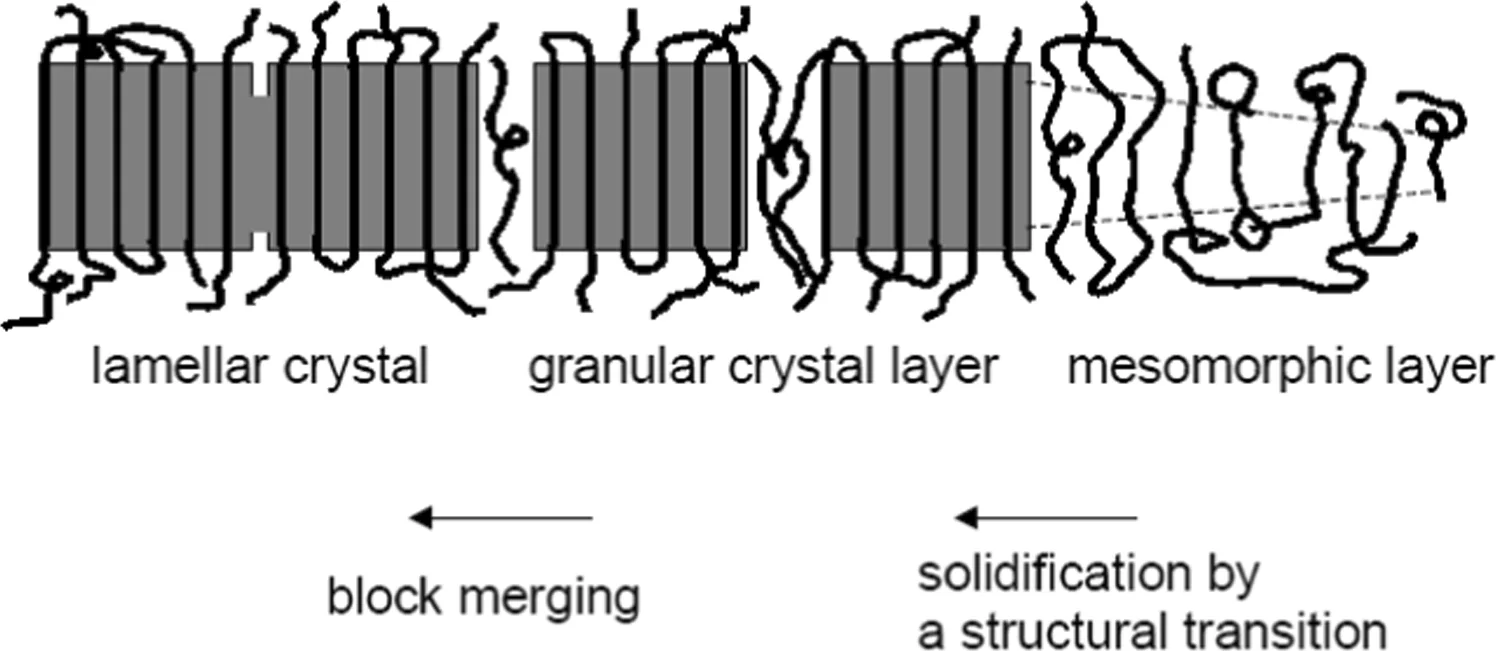
Sketch of the route proposed for the formation of polymer crystallites. Stages passed through as reflected in the structural states found along a layer. Reproduced from [1] with permission from Springer Nature

In 2000, Gert Strobl synthesized a wide range of experimental evidence into a bold and provocative alternative. He proposed that polymer crystallization proceeds not directly from the melt to lamellae, but instead follows a three-stage pathway through a sequence of intermediate states: first, the formation of a mesomorphic layer; second, a cooperative transition into a granular crystalline layer; and finally, the merging and perfection of crystal blocks into a homogeneous lamellar crystallite. This multistep pathway, as illustrated in Fig. 1, challenged the prevailing paradigm and was not a minor modification of existing theory—Strobl’s proposal was nothing short of disruptive; it was a conceptual reframing of the entire process.

The originality of the model lies not only in its structure but also in the breadth of experimental evidence marshaled in its support. Strobl drew on time-resolved SAXS [10–13], AFM [14–16], TEM [17–19], FTIR [20], DSC [21], optical microscopy [22, 23], light scattering [24, 25], dielectric spectroscopy [26], NMR [27], and combined approaches [28, 29] to argue that the melt does not crystallize directly into lamellae. Instead, it passes through transient, partially ordered states that classical models may have overlooked. The mesomorphic layer, with its weak orientational order and slightly elevated density, resembled a liquid-crystalline precursor. The granular crystalline layer, formed through a cooperative transition, echoed two-dimensional ordering phenomena such as the fluid-to-hexatic transition in colloidal monolayers [30, 31]. Moreover, the final lamellae emerged not directly by nucleation at the melt interface but by the merging of nanometer-scale crystal blocks. Later, Strobl supported the original publication [1] with several additional publications [32–40].

This conceptual shift was bold and controversial [3, 5, 41–44]. Nevertheless, Strobl’s model opened up conceptual space for new experiments, new simulations, and new theoretical frameworks. Even if later work refined or refuted certain aspects of the model, the stimulus it provided was profound, as seen with the more than 570 citations in Scopus on 07.08.2026. More than two decades later, Strobl’s model remains influential and continues to be discussed (21 citations in 2025 and 2026 so far). This perspective revisits the conceptual foundations of the path from the mesomorphic to the granular to the lamellar state and examines its implications for modern polymer science. The goal is not to canonize the model, but to clarify why it remains relevant. Strobl’s model was important not because it provided a definitive answer, but because it forced the field to confront its assumptions.

## Retrospective and conceptual origins

### The mesomorphic layer: a liquid-crystalline precursor at the growth front

Strobl’s model begins with the formation of a mesomorphic layer at the interface between the melt and the growing semicrystalline region. This layer is neither fully amorphous nor fully crystalline. Instead, it resembles a weakly ordered liquid-crystalline phase, with chains that are extended but still contain numerous conformational defects. Its density is slightly higher than that of the melt, while its orientational order remains modest, comparable to that found in liquid crystals.

The mesomorphic layer is dynamic and continuously evolving. Chain segments from the melt attach to the growth face, straighten, and integrate into the layer. As they do so, the layer thickens. However, this thickening is not uniform: Mobility is highest at the growth face and decreases with increasing distance from the melt. This mobility gradient is central to the model. It governs the rate of thickening and ultimately triggers the transition to the next structural state.

A key feature of the mesomorphic layer is that it is only stable above a minimum thickness. Below this threshold, the layer would dissolve back into the melt. This minimum thickness sets the starting point for the crystallization pathway. As the layer thickens and its mobility decreases, it eventually reaches a critical thickness, becoming susceptible to a cooperative structural transition.

In placing Strobl’s model into a broader historical context, it is important to recall earlier conceptual developments that anticipated the idea of intermediate states, e.g., [18]. Notably, the concept of a mesomorphic precursor can be traced back to Keller’s mesophase scheme for polyethylene crystallization at wedge-shaped growth fronts. In the early 1990s, Keller and co-workers proposed that, below a crossover temperature, polyethylene crystallizes via a transient hexagonal mesophase that enables rapid, so-called instantaneous lamellar thickening [22, 45]. Despite its conceptual appeal, this hypothesis remained experimentally unsubstantiated.

Strobl later generalized the idea of intermediate states to a wider class of polymers, carefully selecting systems without post-growth thickening—such as syndiotactic polypropylene and polyethylene-co-octene—to minimize annealing effects [33–36, 39, 46–48]. In these materials, he identified a characteristic temperature at which the growth and melting lines converge, corresponding to a limiting lamellar thickness.

### The granular crystalline layer: a cooperative ordering transition

The transition from the mesomorphic layer to the granular crystalline layer is one of the most distinctive and conceptually rich aspects of Strobl’s model. Rather than nucleating isolated crystallites, the mesomorphic layer undergoes a collective transformation. This transition is assumed to be ‘weakly first-order,’ meaning that it involves a discontinuous change in structure but does not require surmounting a significant nucleation barrier. Instead, it resembles the fluid-to-hexatic transition observed in two-dimensional colloidal monolayers or Langmuir films [49].

Strobl’s model can be formalized through a free energy landscape characterized by multiple metastable minima. The mesomorphic layer thickness evolves according to a rate equation that depends on the mobility gradient [37]. The transition from the mesomorphic to the granular crystalline state occurs when the layer reaches a critical thickness *d*_c_, determined by the condition:

$$ G_{{{\mathrm{meso}}}} \left( {d_{{\mathrm{c}}} } \right) = G_{{{\mathrm{granular}}}} \left( {d_{{\mathrm{c}}} } \right) $$

where Δ*G*_meso_ and Δ*G*_granular_ are the Gibbs free energies per unit volume of the respective states. Strobl characterized this transition as ‘weakly first-order’ [1, 32], meaning that it involves a discontinuous change in density and orientational order but proceeds cooperatively without a significant nucleation barrier. This behavior parallels the fluid-to-hexatic transition described by the Kosterlitz–Thouless–Halperin–Nelson–Young theory [49].

In such systems, ordering proceeds through the formation of domains with a preferred orientation, separated by disordered boundary regions. The transition is cooperative: large regions of the system reorganize simultaneously, driven by local density fluctuations and changes in interaction forces. Strobl argued that the mesomorphic layer behaves similarly. As its thickness increases and mobility decreases, density fluctuations amplify, leading to the formation of nanometer-scale crystal blocks arranged in a planar assembly.

The final lamellar state has the lowest free energy Δ*G*_lamella_(*d*_c_) < Δ*G*_granular_(*d*_c_), but the lamellar thickness remains fixed at *d*_c_ during the block merging process, as the perfection process improves internal order without changing macroscopic dimensions. This is a key distinction from the Hoffman–Lauritzen model, where lamellar thickness is determined by a kinetic nucleation barrier that depends on undercooling. A thermodynamic framework for understanding such multistep transitions in polymers has been further developed by Allegra and colleagues, who analyzed mesophase stability through free energy comparisons between liquid, mesomorphic, and crystalline states [50]; see also the discussion in [51]. Keller et al. have discussed the thermodynamic competition between the melting enthalpy and the surface free energy in the nucleation barrier at the early stage of lamellar crystal growth [52]. When the lamella thickness is small, the surface free energy can exceed the melting enthalpy and thus temporarily favor the mesophase (a conformationally disordered hexagonal crystal phase of polyethylene under high pressure) before thickening into the orthorhombic crystal phase occurs. He referred to this as the size factor in phase transitions.

**Fig. 2:**
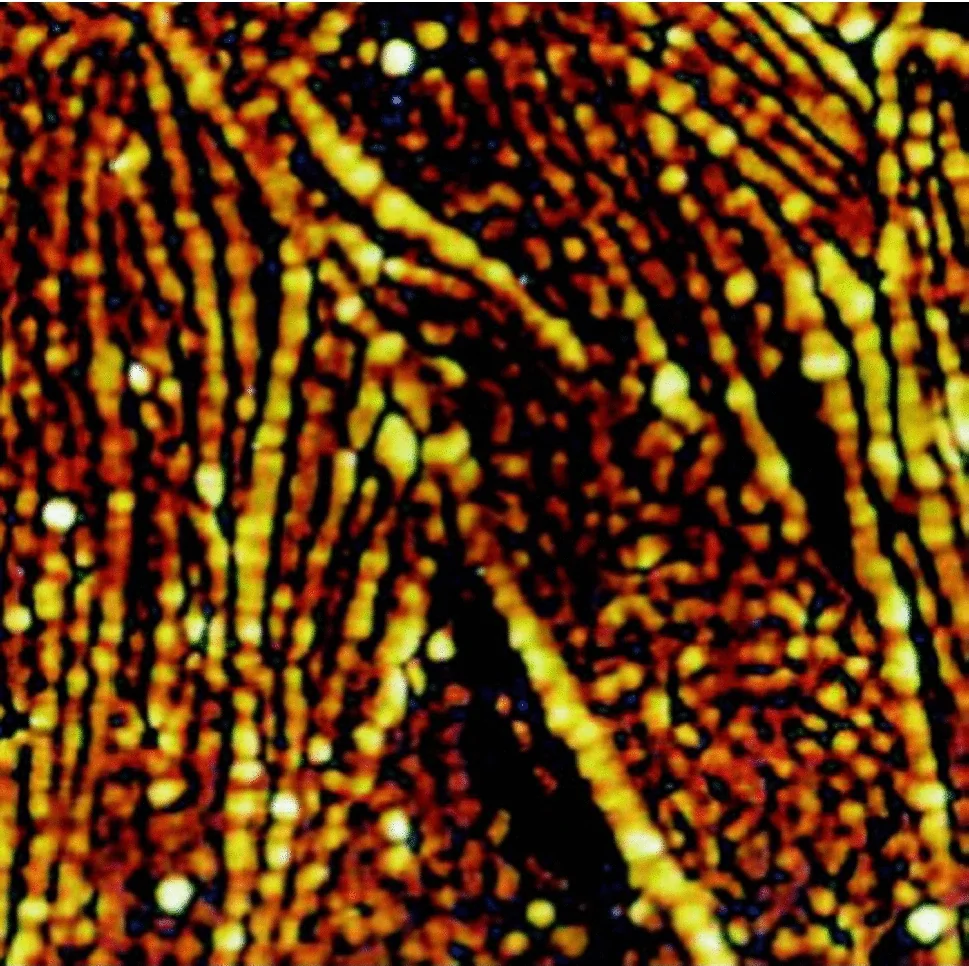
Sample of sPP: AFM tapping mode image of the edges of up standing crystals showing a granular substructure (scan over 1.25 µm in both directions). Reproduced from [39] with permission from the American Physical Society

This granular crystalline layer is neither a lamella with defects nor an accumulation of independently nucleated crystallites. It is a structurally distinct intermediate state, produced by a cooperative transition spanning the entire mesomorphic layer. The blocks have a characteristic size determined by the mesomorphic layer’s critical thickness. Figure 2 shows a typical example of the blocky structure of lamellae.

Beyond the collected experimental evidence, two other specific lines of inquiry likely inspired the “granular” aspect of Strobl’s model. First, the thermodynamic theory of granular nucleation, developed by Giuseppe Allegra, provided a formal basis for treating crystallization as a process involving discrete, nanoscale bundles [50]. Second, work within Strobl’s group at the University of Freiburg on the fracture mechanisms of lamellar crystals revealed a subtle, block-like mosaic structure that emerges at the early stages of deformation [53]. Strobl plausibly connected this mosaic texture, generated mechanically during fracture, to the original growth process of the lamellae, hypothesizing that such a structure might be a remnant of their formation. These developments, combined with his broader experimental program, culminated in the granular crystalline layer as a key intermediate in his multistep model.

The granular crystalline state is metastable. It is more ordered than the mesomorphic layer but less ordered than a fully developed lamella. Its free energy is lower than that of the mesomorphic layer but higher than that of the final lamellar state. This energetic hierarchy drives the next step in the pathway.

### Block merging and perfection: the emergence of lamellar crystallites

The final step in Strobl’s pathway is the merging and perfection of the crystal blocks within the granular crystalline layer. This process reduces the Gibbs free energy and produces a homogeneous lamellar crystallite. Importantly, the lamellar thickness is determined at the moment the mesomorphic layer reaches its critical thickness. The subsequent block merging process improves the internal order of the lamellae but does not alter thickness. However, in certain polymers—specifically crystal-mobile systems—additional post-growth stabilization can occur, leading to modifications of lamellar stability without fundamentally redefining the initial thickness selection.

The resulting lamella retains a subtle mosaic block structure, with thin grain boundaries marking the former block interfaces. These boundaries are typically too small to be detected by conventional scattering techniques but can influence mechanical properties, melting behavior, and recrystallization dynamics.

### Conceptual implications: a shift in how we understand polymer crystallization

Strobl’s model challenges several long-held assumptions in polymer crystallization theory. It suggests that crystallization is not a single-step event but a multistep pathway involving continuous thickening, cooperative transitions, and subsequent perfection. This point of view aligns polymer crystallization with broader concepts in soft matter physics, where metastable intermediates and multistep pathways are common (e.g., Ostwald’s rule of stages).

One of the most significant implications concerns the temperature dependence of growth rates. In the Hoffman–Lauritzen framework, growth rates decrease with increasing temperature because the secondary nucleation barrier increases. In Strobl’s model, growth is controlled by the rate of mesomorphic layer thickening [37]. The critical thickness is reached more slowly at higher temperatures, leading to a decrease in growth rate. This explanation does not rely on nucleation barriers and offers a different interpretation of experimental data.

**Fig. 3:**
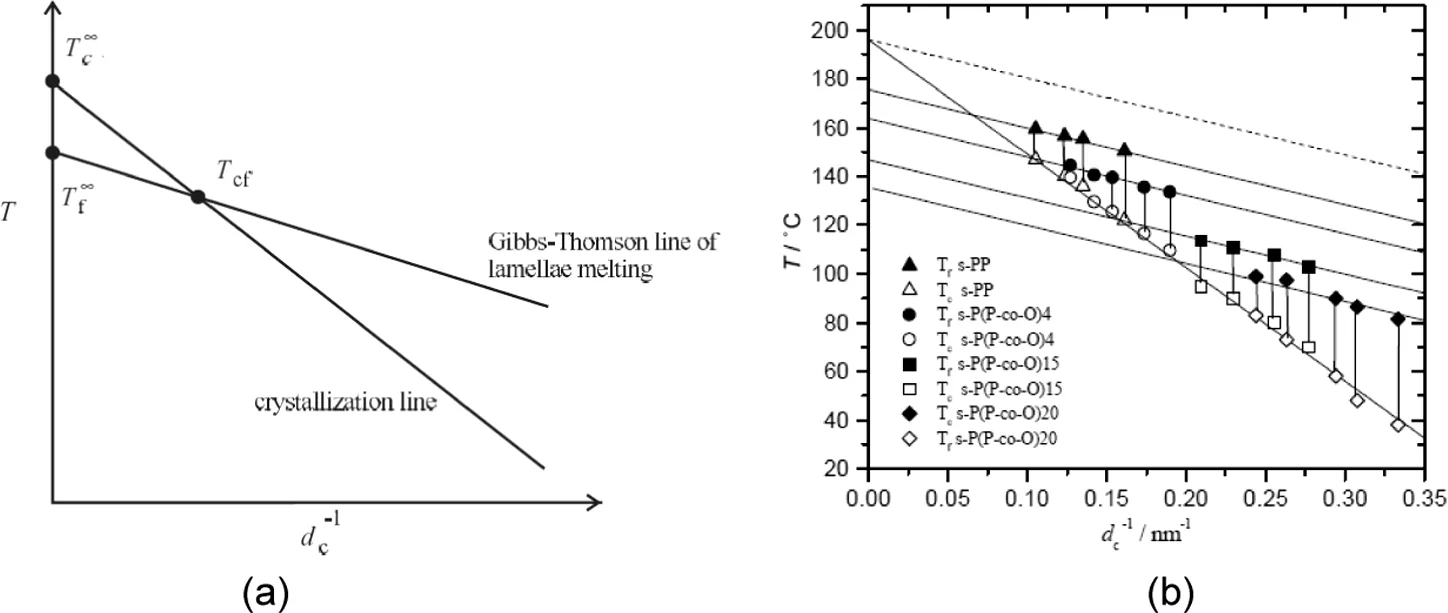
**a** General form of *d*_c_^−1^ versus temperature diagram of crystallizing polymers, set-up of a crystallization and a melting line crossing each other at a finite *d*_c_^−1^. Reproduced from [1] with permission from Springer Nature. **b** s-PP and s-P(P-co-O)x: crystallization line *T*_c_ versus *d*_c_^−1^ (open symbols) and Gibbs–Thomson melting lines *T*_f_ versus *d*_c_^−1^ (filled symbols) as derived from time- and temperature-dependent SAXS experiments. The dotted line represents the extrapolated melting line of perfect s-PP. Reproduced from [32] with permission from Elsevier. The model also highlights the role of mobility gradients in governing crystallization. As the mesomorphic layer thickens, mobility decreases, triggering the cooperative transition. This dynamic perspective shifts the focus from thermodynamic stability to kinetic constraints and dynamic arrest

Another important implication is that lamellar thickness (*d*_c_) is determined early in the process, at the moment the mesomorphic layer reaches its critical thickness. Subsequent perfection does not change the thickness. To support this view, Strobl selected polymers without post-growth thickening to avoid the influence of annealing effects, such as sPP and polyethylene-co-octene. In these samples, he observed a temperature corresponding to a particular lamellae thickness at which the growth line and the melting line intersect, see Fig. 3.

## State of the field—how the paper is relevant today

The distinction between crystal-fixed and crystal-mobile polymers has emerged as a fundamental concept in polymer science, a distinction that was not considered by Strobl when the EPJE paper was published.

Crystal-mobile polymers (e.g., polyethylene, poly(ε-caprolactone), poly(ethylene oxide)) exhibit an α_c_-relaxation process, which indicates that chain segments can undergo translational and rotational motions within the crystalline lattice even below the melting temperature. This intracrystalline mobility enables post-growth thickening and structural relaxation. In crystal-fixed polymers (e.g., polyamide 6, isotactic polystyrene, and poly(butylene succinate)), such mobility is absent, and the crystal structure is kinetically trapped [54–56].

The presence or absence of the α_c_-relaxation has profound consequences for crystallization behavior: Crystal-mobile polymers can undergo thickening of lamellae during or after growth, leading to the stabilization processes Strobl described. In crystal-fixed polymers, such post-growth modifications are prevented, and the crystallization behavior more closely follows the expectations of classical crystallization theories [56–58].

This distinction reinterprets Strobl’s findings: The stabilization he observed is real, but applies only to a subset of polymers and does not necessarily require a mesophase to explain it.

More than two decades later, Strobl’s model remains influential, even though the field has evolved beyond some of its specific assumptions. At the same time, the field has also produced results that challenge key elements of the original model. The intermediate states he proposed—particularly mesomorphic precursors—have not been conclusively confirmed [59].

MD simulation results showing precursor states have been reported by several groups, e.g., [60–66]. Wang et al. demonstrated the existence of medium order structures at the growth front using the site order parameter (SOP) analysis [61], which revealed that the nucleation relies on crystallinity fluctuations with increasing amplitude and that intermediate structures exist unevenly in the early stage of nucleation. Rutledge et al. conducted molecular dynamics simulations to observe the initial process of primary crystal nucleation in long-chain alkanes and were able to observe nuclei within a narrow time window that were suitable for description with the fringed-micelle model [66]. Luo and Sommer performed molecular dynamics simulations to study the growth of a single polymer crystal [60] and entanglement effects on both nucleation and crystal growth [65]. The topological constraints of long-chain polymers were highlighted. Lacevic, Fried, and Gee [62] performed extensive MD simulations of deeply quenched polyethylene melts, identifying mesomorphic domains through clustering algorithms and observing chain sliding diffusion within ordered domains that is related to the ‘chain sliding diffusion’ mechanism suggested by Hikosaka [67]. However, these precursor states differ from the specific mesomorphic layer Strobl proposed, and their relationship to the granular crystalline state remains to be established.

No other mesomorphic states have been experimentally confirmed, aside from the examples Strobl mentioned in the 2000 EPJE paper. One of the strongest indications, namely the difference between crystallization and melting, as revealed in the crystallization and melting lines in Gibbs–Thomson plots, has proven to be a special case only valid for crystal-mobile polymers. In crystal-mobile polymers, chains can move within the crystals, allowing post-growth thickening. For crystal-fixed polymers, such processes are prevented [54–58, 68].

Even this critique goes to the heart of one of Strobl’s central arguments: the separation between crystallization and melting lines in Gibbs–Thomson plots. Strobl interpreted this separation as evidence for an intermediate granular state that stabilizes during crystallization. However, fast-scanning calorimetry [69]—unavailable to Strobl at the time—revealed that for crystal-fixed polymers, the melting temperature differs only minimally from the crystallization temperature [70, 71]. The apparent separation observed in earlier SAXS-based analyses was, in retrospect, an artifact arising from continuous stabilization/reorganization during heating to the melt. Nevertheless, publications continue to appear, showing the predicted behavior with distinguishable crystallization and melting lines [72, 73] or the possible presence of mesophases in early stages of polymer crystallization [74–76].

Strobl selected three different polymers: s-poly(propene-co-octene), poly(ethylene-co-octene), and poly(ε-caprolactone). He recognized that intracrystalline mobility is key to understanding the crystal growth mechanism. Nevertheless, polyethylene exhibits significant intracrystalline mobility, leading to substantial post-growth thickening. The Hoffman–Lauritzen model conventionally compares the post-growth thickness of polyethylene crystals with the theoretical thickness at the growth front, thereby not considering the same stages of crystal growth.

Monte Carlo simulations of lattice polymer crystallization were performed to reproduce Strobl’s experimental observation on the temperature dependence of the excess lamellar thickness. Due to the fixed lattice spacing, the lattice model simulations of polymer crystallization were unable to account for a mesophase at the growth front of lamellar crystals. However, they did reproduce Strobl’s experimental observations regarding the dependence of crystallization and melting temperatures on the reciprocal thickness [77]. The results contradicted the predictions of the Hoffman–Lauritzen theory. This suggests that Strobl conducted a critical experiment to disprove the Hoffman–Lauritzen model, although the mesophase hypothesis appears unnecessary in lattice polymer simulations [77, 78].

Simulation studies have provided complementary insights into polymer crystallization mechanisms beyond the lattice Monte Carlo approaches mentioned above. Rutledge and co-workers have extensively investigated polymer crystallization using atomistic molecular dynamics simulations; see, e.g., [66, 79]. Their work has elucidated the molecular level details of homogeneous crystal nucleation in polyethylene and the role of chain orientation and conformational entropy in the nucleation process. They demonstrated that chain connectivity and entanglement constraints significantly influence the nucleation pathway, with orientational ordering preceding density ordering in some cases.

Sommer and co-workers [60, 80] have employed both MD and Monte Carlo simulations to examine growth pathways, precursor states in single lamellar crystallization, and the interplay between chain topology and crystal growth. Their site order parameter analysis revealed that intermediate structures with medium order exist at the growth front, and that nucleation proceeds through crystallinity fluctuations that increase in amplitude as the critical nucleus forms. These simulations have also elucidated the role of chain sliding diffusion in the thickening of lamellar crystals.

However, these simulation studies face several limitations when testing Strobl’s specific hypothesis. First, the timescales accessible to atomistic MD simulations (typically nanoseconds to microseconds) are far shorter than experimental crystallization times, making it challenging to observe the slow cooperative transitions Strobl proposed. Second, while coarse-grained and lattice models can access longer timescales, they inevitably sacrifice molecular detail regarding conformational order and mobility gradients—features central to Strobl’s model. Third, direct observation of the proposed granular crystalline state requires nanometer-scale spatial resolution that is difficult to achieve in simulations without specialized analysis techniques [81, 82]. Despite these limitations, simulation results generally sought to investigate the concept of multistep pathways in polymer crystallization, even though the specific sequence “mesomorphic→granular→lamellar” remains to be fully validated.

Nevertheless, AFM investigations on the molecular level give some hints for the existence of a mesomorphic structure before nucleation [83] or at the tip of a growing lamella [84].

The broader lesson is that Strobl’s model catalyzed two decades of research that both confirmed and corrected its assumptions. It provided a conceptual framework that motivated new experiments, new simulations, and new theoretical developments. Even where later work contradicted specific elements—such as the necessity of a mesophase—the process of testing and refining the model advanced the field.

## Future directions—where the field is going

Despite significant progress, many questions remain open. The molecular origin of early stage ordering is still under debate. In some polymers, pre-ordering resembles a hexagonal or liquid-crystalline phase; in others, it appears as a density-modulated precursor without clear orientational order. Understanding these differences will require advanced scattering techniques, spectroscopic probes, and simulations capable of resolving such subtle structural features.

The factors determining lamellar thickness also remain an active area of research. Mobility gradients, entanglement constraints, interfacial energies, and local packing frustration likely all contribute. Developing a predictive theory that integrates these effects remains a major challenge.

The universality of granular crystalline states is another open question. While they appear in many systems, their presence and stability depend sensitively on cooling rate, molecular architecture, and processing conditions.

The key challenges for future experimental and simulation studies include:

1. Time-resolved observation of early stage ordering: Techniques capable of probing structural evolution on the timescale of mesomorphic layer formation (milliseconds to seconds) are essential. This could include:
  In situ fast-scanning calorimetry coupled with simultaneous SAXS/WAXS to correlate thermal events with structural changes [69]. Recent work has demonstrated that FSC samples can be analyzed by microfocus WAXS [85, 86], TEM [87], or optical microscopy [88], enabling direct structure analysis of samples with well-defined thermal histories.
  Ultra-fast X-ray scattering at synchrotron sources with millisecond time resolution [89, 90].
  High-speed AFM at elevated temperatures to directly image growth front dynamics [83, 84].
2. Direct detection of the granular crystalline state: This requires distinguishing nanometer-scale block structures from conventional lamellae with defects. Promising approaches include:
  Advanced microscopy with image processing to reveal mosaic structures [91–93].
  Scanning nano-diffraction (e.g., ptychography) to map local crystallinity with nanometer resolution [94, 95].
  Solid-state NMR with isotopic labeling to probe local chain organization [58].
3. Testing the universality of the pathway: Systematic studies across different polymer classes (crystal-fixed vs. crystal-mobile) under identical conditions would clarify whether the multistep pathway is universal or polymer-dependent. Recent work on poly(ethylene brassylate) showing crystallization rate minima at transitions between quantized crystal thicknesses provides an example of how detailed kinetic studies can reveal stepwise growth mechanisms [96].
4. Simulations that bridge timescales: Hybrid approaches combining atomistic MD for short-timescale conformational dynamics with coarse-grained or Monte Carlo methods for long-timescale ordering, coupled with machine learning to identify emergent intermediate states [81, 82], could provide a comprehensive picture.

Finally, data-driven approaches offer new opportunities. By analyzing large datasets from simulations and experiments, it may be possible to identify universal features of early stage ordering, classify hidden intermediate states, and predict crystallization pathways under different conditions [97, 98].

## Conclusion

Strobl’s 2000 EPJE paper proposed a bold reimagining of polymer crystallization. By introducing mesomorphic and granular crystalline layers as essential intermediates, it shifted the field’s attention from nucleation barriers to mobility gradients, cooperative transitions, and multistep pathways. More than two decades later, far from being universally accepted, the model remains influential because it continues to stimulate new experiments, new theories, and new ways of thinking. It fundamentally changed how the field approaches crystallization, encouraged researchers to look beyond the lamella and to consider the complex pathways leading to it.

In many ways, the field is still catching up to the conceptual shift Strobl initiated. As advanced characterization techniques, high-resolution simulations, and data-driven analyses continue to advance, revisiting the mesomorphic–granular–lamellar pathway remains a promising direction. Regardless of whether the model proves to be universally applicable or serves as a stepping stone toward an even more comprehensive framework, its legacy is secure: it has challenged polymer scientists to look beyond the lamella and to explore the complexity of the pathways that lead to it.

## Data Availability

The manuscript has no associated data.
